# COVID-19 and neuropathy in type 2 diabetes

**DOI:** 10.1038/s41598-025-95133-4

**Published:** 2025-04-01

**Authors:** Georgios Ponirakis, Ibrahim Al-Janahi, Einas Elgassim, Aisha Al Obaidan, Hoda Gad, Ioannis N. Petropoulos, Adnan Khan, Hadeel B. Zaghloul, Hamda Ali, Mashhood A. Siddique, Fatima F. S. Mohamed, Lina H. M. Ahmed, Youssra Dakroury, Abeer M. M. El Shewehy, Shaikha N. Al-Thani, Farheen Ahmed, Rawan Hussein, Salah Mahmoud, Iuliia Salivon, Moayad Homssi, Nebras H. Hadid, Ateeque Mohamed Ali, Safah Khan, Ziyad R. Mahfoud, Mahmoud A. Zirie, Gulfidan Bitirgen, Yousuf Al-Ansari, Mohammed I. Alhatou, Stephen L. Atkin, Rayaz A. Malik

**Affiliations:** 1 Department of Medicine, Weill Cornell Medicine-Qatar, Qatar Foundation, Doha, Qatar; 2 National Diabetes Center, Hamad General Hospital, Hamad Medical Corporation, Doha, Qatar; 3 Division of Cardiovascular Sciences, Faculty of Biology, Medicine and Health, University of Manchester, Manchester, UK; 4 Department of Ophthalmology, Necmettin Erbakan University Meram Medical Faculty Hospital, Konya, Turkey; 5 Department of Neurology, Hamad General Hospital, Hamad Medical Corporation, Doha, Qatar; 6 Royal College of Surgeons in Ireland Bahrain, Adliya, Kingdom of Bahrain; 7 Faculty of Science and Engineering, Manchester Metropolitan University, Manchester, UK; 8 Weill Cornell Medicine-Qatar, Qatar Foundation, Education City, PO Box: 24144, Doha, Qatar

**Keywords:** COVID-19, Diabetic peripheral neuropathy, Type 2 diabetes, Corneal confocal microscopy, Severe COVID-19, long-COVID, Corneal nerve measures, Biomarkers, Diseases, Endocrinology, Medical research, Neurology, Risk factors

## Abstract

This study investigated the risk factors for COVID-19 and its impact on diabetic peripheral neuropathy (DPN) in patients with type 2 diabetes (T2D). Patients with T2D underwent assessments with the NICE post-COVID questionnaire, DN4 questionnaire, vibration perception threshold (VPT), and corneal confocal microscopy (CCM) before and 11.0 ± 8.9 months after developing COVID-19. Of 76 participants with T2D, 35 (46.1%) developed COVID-19, of whom 8 (22.9%) developed severe COVID-19 and 9 (25.7%) developed long-COVID. The development of COVID-19 was associated with lower systolic blood pressure (*P* < 0.05). The presence and severity of DPN were not associated with developing COVID-19, severe COVID-19, or long-COVID (*P* = 0.42–0.94). Women were eight times more likely to develop long-COVID (*P* < 0.05) and elevated body weight, LDL, and VPT were associated with the development of long-COVID (*P* < 0.05 − 0.01). The long-COVID group exhibited significant changes in triglycerides and LDL (*P* < 0.05 for both) and body weight (*P* < 0.01) at follow-up. Their impact on clinical and neuropathy measures was comparable in patients with and without COVID-19 (*P* = 0.08–0.99). There was a significant reduction in corneal nerve measures (*P* < 0.05-0.0001) in patients with and without COVID-19. A low systolic blood pressure, altered lipids, body weight, higher VPT, and gender may determine the impact of COVID-19 in patients with T2D, but there was no evidence of an impact of COVID-19 on the development or progression of DPN.

**Supplementary Information:**

The online version contains supplementary material available at 10.1038/s41598-025-95133-4.

## Introduction

It has been proposed that patients with diabetes, renal, and chronic pulmonary diseases^1^ as well as diabetic peripheral neuropathy (DPN)^2^ may have a higher risk of developing severe COVID-19. This is of concern, given that globally 10.5% of adults have T2D^3^ and at least 50% will develop DPN^4^.

Severe acute respiratory syndrome coronavirus 2 (SARS-CoV-2) infection is associated with a range of neurological manifestations including fatigue, cognitive impairment, and loss of sense of smell and taste^5^. Post-COVID-19 neuropathy, particularly small fiber neuropathy has been attributed to direct nerve damage caused by the virus, immune-mediated neurodegeneration, or a combination of both^6–8^. There are reports of worsening neurological disability in several neuromuscular conditions following SARS-CoV-2 infection^9–13^ and the development of neuropathy^2,14,15^. We have previously shown that severe COVID-19 was associated with loss of taste, smell, and neuropathic pain with abnormal thermal thresholds in patients with type 2 diabetes (T2D)^15^. Furthermore, intraepidermal nerve fiber^7^ and corneal nerve fiber^8^ loss have been reported in patients with long-COVID^16^.

This study sought to determine whether pre-existing clinical and metabolic factors and presence as well as severity of neuropathy increased the risk of developing COVID-19, severe COVID-19, or long-COVID and whether COVID-19 worsens DPN in patients with T2D.

## Methods

### Project design

Participants with type 2 diabetes (T2D) were initially recruited in January 2017 as part of a longitudinal study investigating diabetic neuropathy at the National Diabetes Center, Hamad General Hospital, Qatar. Following the onset of the COVID-19 pandemic, data from this pre-existing cohort was used to investigate the impact of COVID-19 on neuropathy outcomes. Ethical approval was obtained for both the longitudinal study (#14–00058, valid from 29/10/2016 to 28/10/2024) and the secondary COVID study (#20–00024) from the Institutional Review Boards of Weill Cornell Medicine-Qatar (WCM-Q IRB) and Hamad Medical Corporation (HMC IRB) under reference numbers #15,103/15 and MRC-01-21-386, respectively, in compliance with the Declaration of Helsinki. Informed consent was obtained from all participants before their inclusion in the study. All methods were performed in accordance with the relevant guidelines and regulations.

The study design included two assessment periods: the first conducted in January 2017 prior to the COVID-19 pandemic, and the second in October 2022 following the pandemic. The COVID-19 group included participants who tested positive for COVID-19 via RT-PCR during the study period, while the no-COVID group consisted of participants from the original cohort who did not contract COVID-19, as confirmed through medical records and RT-PCR testing. Both groups were assessed during the same follow-up period in October 2022 to ensure consistency in timing and methodology. The COVID-19 group was further classified into mild cases (COVID group excluding severe COVID and long-COVID), those hospitalized with pneumonia (severe COVID group), and those with symptoms persisting for at least four weeks after infection (long-COVID group). We excluded patients with severe COVID-19 and long-COVID from the COVID group to ensure there were no confounding effects of more severe and sustained disease. The severe COVID group did not include individuals with long-COVID, and vice versa.

This research included individuals aged between 18 and 80 years diagnosed with T2D. Exclusion criteria were severe chronic dry eye syndrome, corneal dystrophy, any history of ocular injury or surgery within the preceding 12 months, or an allergy to Oxybuprocaine, a local anesthetic used in confocal corneal microscopy (CCM). Additional exclusions included renal failure (chronic kidney disease stages 4 and 5), ongoing corticosteroid therapy, active retinopathy, pregnancy, and neuropathy not related to diabetes, such as Sjogren’s syndrome, systemic lupus erythematosus, HIV, hepatitis B and C, inherited neuropathies, chemotherapy-induced neuropathy, and alcohol misuse.

### Independent variables

*Post-COVID syndrome questionnaire* – The Post-COVID NICE questionnaire was developed in-house to assess neurological and systemic symptoms post-COVID-19 and has been used previously by Bitirgen et al. (2021)^16^. This questionnaire consists of questions on pre-morbid medical conditions, severity of COVID-19 infection, and a range of COVID-19 related symptoms: 4 weeks before COVID-19, 4 weeks after initial COVID-19 symptoms or testing positive, and 4 weeks prior to assessment. The main symptoms assessed included fatigue/tiredness, brain fog, memory or concentration loss, headache, loss or change to sense of taste/smell, pins and needles/numbness, dizziness, breathlessness, cough, sore throat, chest pain, abdominal/stomach pain, nausea/feeling sick, and diarrhea.

Long-COVID was defined by the presence of fatigue, brain fog, memory or concentration loss, loss or change in the sense of taste or smell that developed after COVID-19 and persisted for at least 4 weeks.

Participants in the no COVID group were asked to respond to the symptom questions at: (1) 4 weeks before COVID-19 (baseline reference period) and (2) 4 weeks prior to assessment (reflecting their current health status). For participants in the COVID, severe COVID, and long-COVID groups, they were asked to respond to the symptom questions at: (1) 4 weeks before COVID-19 (pre-infection baseline), (2) 4 weeks after initial COVID-19 symptoms or tested positive (acute phase of the infection), and (3) 4 weeks prior to assessment (reflecting their current health status).

### Assessment of neuropathy (dependent variables)

The evaluation of neuropathic symptoms and signs was conducted using the Douleur Neuropathique en 4 (DN4) questionnaire, which involves an evaluation of seven symptoms including sensation of burning, cold pain, electric shocks, tingling, pins and needles, numbness, and itching^17,18^. Additionally, it assesses three physical signs: reduced sensitivity to touch, diminished response to pinprick, and allodynia.

The vibration perception threshold (VPT) was assessed at the pulp of the large toe using a Neurothesiometer (Horwell, Scientific Laboratory Supplies, Wilford, Nottingham, UK) and the mean of three readings was documented.

Corneal confocal microscopy (CCM) assessments were carried out utilizing the Heidelberg Retina Tomograph equipped with the Rostock Cornea Module (Heidelberg Engineering GmbH, Heidelberg, Germany)^19^. To ensure patient comfort during the procedure, Oxybuprocaine hydrochloride 0.4% (Chauvin Pharmaceuticals, Chefaro, UK) was applied as a local anesthetic to the eyes. The interface between the cornea and the CCM cap was established using Viscotears gel (Carbomer 980, 0.2%, Novartis, UK). During the examination, participants were asked to focus on a specific point with the eye that was not being examined to capture multiple images of the sub-basal nerve plexus in the central cornea. The field of view of the captured image is 400 × 400 μm. Later, a researcher, not aware of the participants’ status, chose three high-quality images for each eye^20^. The selection criteria focused on image depth, clarity of focus, and contrast. Manual analysis of the corneal nerve fiber density (CNFD) (main nerve fibers/mm^2^), branch density (CNBD) (branches/mm^2^), and fiber length (CNFL) (length of main nerves and branches in mm /mm^2^) were performed using the CCMetrics software^21^.

Diabetic peripheral neuropathy (DPN) was diagnosed^22^ based on ≥ 4 neuropathic symptoms, including numbness, burning pain, electric shocks, tingling, pins and needles, painful cold, and a VPT ≥ 15 V in the feet or CNFL ≤ 17 mm/mm^2^.

### Clinical and metabolic measures (covariates)

These include age, diabetes duration, BMI, systolic (SBP) and diastolic (DBP) blood pressure, HbA1c, and lipid profile.

### Data analysis and statistics

Data is presented as the mean and standard deviation. Overall differences between groups (among individuals with and without COVID-19, those with severe COVID-19, and patients experiencing long-COVID) were assessed using a one-way Analysis of Variance (ANOVA) with post-hoc analysis between individual groups using the Least Significant Difference (LSD) method. ANOVA was conducted using only existing data, without imputing values for any missing data points. Logistic regression was used to evaluate the association between COVID-19 status and DPN, while linear regression was used to assess the effect of COVID-19 on changes in neuropathy measures. All statistical analyses were performed using IBM-SPSS software (version 29, SPSS Inc., Armonk, NY). Statistical significance was established at a two-tailed P-value of 0.05 or less.

## Results

76 participants with type 2 diabetes (T2D) underwent assessment in the pre- and post-COVID-19 period. Of these 76 participants, 35 (46.1%) had COVID-19, of whom 8 (22.9%) had severe COVID-19 and 9 (25.7%) developed long-COVID. Loss of taste or smell occurred immediately in 10 (28.6%) and remained in 2 (5.7%), fatigue occurred immediately in 15 (42.9%) and remained in 5 (14.3%), and brain fog or memory issues occurred immediately in 4 (11.4%) and remained in 5 (14.3%). Participants underwent follow-up assessments 11 ± 8.9 months after the onset of COVID-19.

### Differences in clinical and neuropathy measures between T2D patients with and without COVID-19

Before COVID-19 infection, age, sex, body weight, BMI, HbA1c, total cholesterol, LDL, HDL, triglycerides, diastolic blood pressure (DBP), prevalence of DPN, neuropathic symptoms, vibration perception threshold (VPT) and corneal nerve morphology were comparable and only systolic blood pressure (SBP) was significantly lower (118.4 ± 17.8 vs. 129.1 ± 16.2, *P* < 0.05) in the COVID-19 group (excluding those with severe COVID and long-COVID) (*n* = 21/35, 60.0%) compared to those who did not develop COVID-19 (Tables 1 and 2).

**Table 1:** Comparison of pre-COVID, post-COVID, and changes in clinical characteristics between participants with type 2 diabetes without COVID infection, and mild and severe COVID.

	No COVID-19 (*n* = 41)	COVID-19 (*n* = 21)	Severe COVID-19 (*n* = 8)	*P* value
Age, years	54.9 ± 9.0	56.2 ± 6.1	55.7 ± 7.3	0.89
Men, n (%)	21 (70.0)	5 (16.7)	4 (13.3)	0.14
Women, n (%)	20 (51.3)	15 (38.5)	4 (10.3)	
Hb1Ac pre-COVID, %	8.2 ± 1.0	8.1 ± 0.9	8.4 ± 1.1	0.47
Hb1Ac post-COVID, %	8.1 ± 1.4	8.0 ± 1.2	8.5 ± 1.8	0.38
ΔHb1Ac, %	-0.1 ± 1.3	-0.1 ± 1.5	0.2 ± 1.4	0.52
Total cholesterol pre-COVID, mmol/L	4.18 ± 1.19	3.88 ± 0.87	4.67 ± 1.09	0.35
Total cholesterol post-COVID, mmol/L	3.99 ± 1.24	4.28 ± 0.63	4.56 ± 1.11	0.63
ΔTotal cholesterol, mmol/L	-0.19 ± 1.51	0.40 ± 0.95	-0.11 ± 1.22	0.38
Triglyceride pre-COVID, mmol/L	1.81 ± 1.27	1.68 ± 1.32	1.11 ± 0.20	0.30
Triglyceride post-COVID, mmol/L	1.50 ± 0.68	1.46 ± 0.74	1.61 ± 1.18	0.66
ΔTriglyceride, mmol/L	-0.34 ± 1.26	-0.25 ± 1.56	0.50 ± 1.16	0.21
HDL pre-COVID, mmol/L	1.07 ± 0.28	1.09 ± 0.28	**1.37 ± 0.33†**	**< 0.05**
HDL post-COVID, mmol/L	1.20 ± 0.34	1.35 ± 0.45	1.46 ± 0.35	0.51
ΔHDL, mmol/L	0.13 ± 0.26	0.26 ± 0.39	0.09 ± 0.12	0.21
LDL pre-COVID, mmol/L	2.28 ± 0.87	2.00 ± 0.70	**2.84 ± 0.98**	**< 0.05**
LDL post-COVID, mmol/L	1.94 ± 0.87	2.33 ± 0.55	2.41 ± 0.90	0.80
ΔLDL, mmol/L	-0.32 ± 0.80	0.33 ± 0.71**†**	-0.43 ± 0.96	**<** **0.05**
Systolic BP pre-COVID, mmHg	129.1 ± 16.2	118.4 ± 17.8**‡**	128.0 ± 20.4	0.19
Systolic BP post-COVID, mmHg	134.9 ± 12.9	127.1 ± 14.1**‡**	128.8 ± 15.9	0.77
ΔSystolic BP, mmHg	5.9 ± 19.3	8.7 ± 19.17	0.8 ± 29.1	0.36
Diastolic BP pre-COVID, mmHg	78.4 ± 8.8	74.1 ± 11.4	74.4 ± 12.9	0.94
Diastolic BP post-COVID, mmHg	75.1 ± 7.5	74.7 ± 8.4	73.5 ± 9.5	0.73
ΔDiastolic BP, mmHg	-3.4 ± 12.0	0.6 ± 12.0	-0.9 ± 16.4	0.78
Body weight pre-COVID, kg	84.8 ± 12.0	79.0 ± 10.2	81.0 ± 10.8	0.69
Body weight post-COVID, kg	80.8 ± 11.2	78.2 ± 9.4	83.5 ± 12.3	0.27
ΔBody weight, kg	-4.0 ± 9.5	-0.8 ± 7.8	2.4 ± 5.2	0.39
BMI pre-COVID, kg/m^2^	31.9 ± 2.9	32.1 ± 3.7	30.2 ± 2.9	0.20
BMI post-COVID, kg/m^2^	31.0 ± 3.5	31.5 ± 4.9	31.8 ± 3.6	0.86
ΔBMI, kg/m^2^	-0.9 ± 2.6	-0.6 ± 3.6	1.6 ± 2.3	0.10

Numeric and categorical variables are summarized as means ± standard deviation and n (%), respectively. Variables were compared using one-way ANOVA except for sex which was compared using χ^2^. Variables that were significantly different between the no COVID-19 group and COVID-19 groups were denoted as ‡*P* ≤ 0.05, †*P* ≤ 0.01, ††*P* ≤ 0.001, †††*P* ≤ 0.0001. P value for mild vs. severe COVID. Abbreviations: Δ = difference and BP = blood pressure.

**Table 2:** Comparison of pre-COVID, post-COVID, and changes in neuropathy characteristics between patients with type 2 diabetes without COVID infection, and mild and severe COVID.

	No COVID (*n* = 41)	COVID-19 (*n* = 21)	Severe COVID-19 (*n* = 8)	*P* value
DPN (DN4 & CNFL) pre-COVID, n (%)	5/41 (12.2)	2/21 (9.5)	2/7 (28.6)	0.42
DPN (DN4 & CNFL) post-COVID, n (%)	10/41 (24.4)	5/21 (23.8)	3/8 (37.5)	0.72
DPN (DN4 & VPT) pre-COVID, n (%)	5/40 (12.5)	2/20 (10.0)	1/7 (14.3)	0.94
DPN (DN4 & VPT) post-COVID, n (%)	3/40 (7.5)	2/20 (10.0)	2/8 (25.0)	0.33
CNFD pre-COVID, fibers/mm^2^	26.3 ± 9.5	27.4 ± 9.6	24.4 ± 8.4	0.51
CNFD post-COVID, fibers/mm^2^	22.3 ± 8.3	23.0 ± 7.9	21.5 ± 10.8	0.49
ΔCNFD, fibers/mm^2^	-4.0 ± 6.7	-4.3 ± 8.0	-2.9 ± 8.0	0.97
CNBD pre-COVID, branches/mm^2^	62.9 ± 35.6	72.3 ± 51.3	62.0 ± 31.9	0.55
CNBD post-COVID, branches/mm^2^	40.7 ± 28.6	39.4 ± 25.8	31.8 ± 15.4	0.50
ΔCNBD, branches/mm^2^	-22.2 ± 33.3	-32.9 ± 44.7	-30.2 ± 24.1	0.87
CNFL pre-COVID, mm/mm^2^	17.3 ± 6.4	18.1 ± 6.8	16.4 ± 6.2	0.57
CNFL post-COVID, mm/mm^2^	14.9 ± 6.2	14.6 ± 5.2	13.2 ± 5.3	0.44
ΔCNFL, mm/mm^2^	-2.3 ± 4.8	-3.5 ± 5.0	-3.2 ± 3.9	0.88
VPT pre-COVID, V	9.6 ± 5.7	7.8 ± 4.3	10.5 ± 5.7	0.27
VPT post-COVID, V	9.8 ± 6.9	8.9 ± 5.3	11.6 ± 6.8	0.37
ΔVPT, V	0.2 ± 6.1	1.1 ± 4.0	1.1 ± 2.6	0.98
DN4 pre-COVID, score	2.5 ± 2.7	2.6 ± 2.0	3.3 ± 2.5	0.54
DN4 post-COVID, score	2.2 ± 2.2	2.4 ± 2.4	3.1 ± 2.7	0.46
ΔDN4, score	-0.3 ± 2.2	-0.2 ± 2.2	-0.1 ± 2.7	0.92

Numeric variables are summarized as means ± standard deviation. Variables were compared using one-way ANOVA. Variables that were significantly different between the no COVID-19 group and COVID-19 groups were denoted as ‡*P* ≤ 0.05, †*P* ≤ 0.01, ††*P* ≤ 0.001, †††*P* ≤ 0.0001. P value for mild vs. severe COVID. Abbreviations: Δ = difference, CNFD = corneal nerve fiber density, CNBD = corneal nerve branch density, CNFL = corneal nerve fiber length, and VPT = vibration perception threshold.

### Differences in clinical and neuropathy measures between patients with and without severe COVID-19

In the pre-COVID phase, age, gender, HbA1c, total cholesterol, triglycerides, SBP, DBP, body weight, BMI, prevalence of DPN, neuropathic symptoms, VPT, and corneal nerve morphology did not differ between patients with severe COVID-19, with COVID-19 and without COVID-19. LDL (2.84 ± 0.98 vs. 2.00 ± 0.70, *P* < 0.05) and HDL (1.37 ± 0.33 vs. 1.09 ± 0.28, *P* ≤ 0.05) were significantly higher in the severe COVID-19 group compared to the COVID-19 group (Tables 1 and 2).

### Differences in clinical and neuropathy measures between patients with and without long-COVID

Age, sex, HbA1c, total cholesterol, LDL, triglycerides, DBP, prevalence of DPN, neuropathic symptoms, and corneal nerve morphology did not differ between those with long-COVID compared to those with and without COVID-19. However, the prevalence of long-COVID was ~ 8 fold higher in women (18.6% vs. 3.7%) and body weight was significantly higher compared to the COVID-19 group (91.4 ± 14.8 kg vs. 79.0 ± 10.2 kg, *P* = 0.01) but comparable to the no COVID-19 group (84.8 ± 12.0, *P* = 0.15). HDL (1.32 ± 0.49 vs. 1.057± 0.28, *P* ≤ 0.05), and LDL (2.82 ± 1.07 vs. 2.00 ± 0.70, *P* < 0.05) were significantly higher in the long-COVID group compared to the COVID-19 group. VPT (13.4 ± 6.2 vs. 7.8 ± 4.3, *P* ≤  0.05) was significantly higher in the long-COVID group compared to the COVID-19 group (Tables 3 and 4).

**Table 3:** Comparison of pre-COVID (baseline), post-COVID (last visit), and changes (difference) in clinical characteristics between participants with type 2 diabetes without COVID infection, with COVID, and long-COVID.

	No COVID (*n* = 41)	COVID (*n* = 21)	Long COVID (*n* = 9)	*P* value
Age, years	54.9 ± 9.0	56.2 ± 6.1	51.3 ± 6.8	0.13
Men, n (%)	21 (77.8)	5 (18.5)	1 (3.7)	**< 0.05**
Women, n (%)	20 (46.5)	15 (34.9)	8 (18.6)	
Hb1Ac pre-COVID, %	8.2 ± 1.0	8.1 ± 0.9	8.4 ± 0.7	0.39
Hb1Ac post-COVID, %	8.1 ± 1.4	8.0 ± 1.2	8.9 ± 1.6	0.10
ΔHb1Ac, %	-0.1 ± 1.3	-0.1 ± 1.5	0.5 ± 1.5	0.32
Total cholesterol pre-COVID, mmol/L	4.18 ± 1.19	3.88 ± 0.87	4.86 ± 1.37	0.051
Total cholesterol post-COVID, mmol/L	3.99 ± 1.24	4.28 ± 0.63	4.86 ± 0.99**‡**	0.22
ΔTotal cholesterol, mmol/L	-0.19 ± 1.51	0.40 ± 0.95	0.01 ± 0.86	0.49
Triglyceride pre-COVID, mmol/L	1.81 ± 1.27	1.68 ± 1.32	1.77 ± 0.97	0.87
Triglyceride post-COVID, mmol/L	1.50 ± 0.68	1.46 ± 0.74	2.82 ± 1.86**††**	**0.001**
ΔTriglyceride, mmol/L	-0.34 ± 1.26	-0.25 ± 1.56	1.05 ± 1.83**‡**	**< 0.05**
HDL pre-COVID, mmol/L	1.07 ± 0.28	1.09 ± 0.28	1.32 ± 0.49**‡**	0.09
HDL post-COVID, mmol/L	1.20 ± 0.34	1.35 ± 0.45	1.40 ± 0.48	0.76
ΔHDL, mmol/L	0.13 ± 0.26	0.26 ± 0.39	0.08 ± 0.26	0.19
LDL pre-COVID, mmol/L	2.28 ± 0.87	2.00 ± 0.70	2.82 ± 1.08	**< 0.05**
LDL post-COVID, mmol/L	1.94 ± 0.87	2.33 ± 0.55	2.36 ± 0.97	0.91
ΔLDL, mmol/L	-0.32 ± 0.80	0.33 ± 0.71**†**	-0.46 ± 0.76	< **0.05**
Systolic BP pre-COVID, mmHg	129.1 ± 16.2	118.4 ± 17.8**†**	122.8 ± 11.6	0.50
Systolic BP post-COVID, mmHg	134.9 ± 12.9	127.1 ± 14.1**‡**	122.7 ± 14.1**†**	0.42
ΔSystolic BP, mmHg	5.9 ± 19.3	8.7 ± 19.17	-0.1 ± 12.7	0.25
Diastolic BP pre-COVID, mmHg	78.4 ± 8.8	74.1 ± 11.4	74.7 ± 9.4	0.88
Diastolic BP post-COVID, mmHg	75.1 ± 7.5	74.7 ± 8.4	73.3 ± 8.1	0.68
ΔDiastolic BP, mmHg	-3.4 ± 12.0	0.6 ± 12.0	-1.3 ± 7.3	0.68
Body weight pre-COVID, kg	84.8 ± 12.0	79.0 ± 10.2	91.4 ± 14.8	**0.01**
Body weight post-COVID, kg	80.8 ± 11.2	78.2 ± 9.4	83.1 ± 11.7	0.28
ΔBody weight, kg	-4.0 ± 9.5	-0.8 ± 7.8	-8.3 ± 15.7**†**	**< 0.01**

Numeric and categorical variables are summarized as means ± standard deviation and n (%), respectively. Variables were compared using one-way ANOVA except for sex which was compared using χ^2^. Variables that were significantly different between the no COVID-19, COVID-19, and long-COVID group were denoted as ‡*P* ≤ 0.05, †*P* ≤ 0.01, ††*P* ≤ 0.001, †††*P* ≤ 0.0001. P value for COVID-19 vs. long-COVID. Abbreviations: Δ = difference and BP = blood pressure.

**Table 4:** Comparison of pre-COVID, post-COVID, and changes in neuropathy characteristics between participants with type 2 diabetes without COVID infection, with COVID, and long-COVID.

	No COVID-19 (*n* = 41)	COVID-19 (*n* = 21)	Long-COVID (*n* = 9)	*P* value
DPN (DN4 & CNFL) pre-COVID, n (%)	5/41 (12.2)	2/21 (9.5)	1/9 (11.1)	0.95
DPN (DN4 & CNFL) post-COVID, n (%)	10/41 (24.4)	5/21 (23.8)	3/9 (33.3)	0.84
DPN (DN4 & VPT) pre-COVID, n (%)	5/40 (12.5)	2/20 (10.0)	2/8 (25.0)	0.56
DPN (DN4 & VPT) post-COVID, n (%)	3/40 (7.5)	2/20 (10.0)	2/9 (22.2)	0.42
CNFD pre-COVID, fibers/mm^2^	26.3 ± 9.5	27.4 ± 9.6	24.4 ± 5.3	0.41
CNFD post-COVID, fibers/mm^2^	22.3 ± 8.3	23.0 ± 7.9	25.2 ± 5.5	0.49
ΔCNFD, fibers/mm^2^	-4.0 ± 6.7	-4.3 ± 8.0	0.8 ± 8.0	0.08
CNBD pre-COVID, branches/mm^2^	62.9 ± 35.6	72.3 ± 51.3	50.0 ± 23.4	0.22
CNBD post-COVID, branches/mm^2^	40.7 ± 28.6	39.4 ± 25.8	37.6 ± 20.6	0.88
ΔCNBD, branches/mm^2^	-22.2 ± 33.3	-32.9 ± 44.7	-12.4 ± 28.5	0.21
CNFL pre-COVID, mm/mm^2^	17.3 ± 6.4	18.1 ± 6.8	15.8 ± 4.5	0.36
CNFL post-COVID, mm/mm^2^	14.9 ± 6.2	14.6 ± 5.2	14.9 ± 3.7	0.88
ΔCNFL, mm/mm^2^	-2.3 ± 4.8	-3.5 ± 5.0	-0.9 ± 5.5	0.18
VPT pre-COVID, V	9.6 ± 5.7	7.8 ± 4.3	13.4 ± 6.2	< **0.05**
VPT post-COVID, V	9.8 ± 6.9	8.9 ± 5.3	15.0 ± 7.6	0.07
ΔVPT, V	0.2 ± 6.1	1.1 ± 4.0	1.6 ± 2.6	0.87
DN4 pre-COVID, score	2.5 ± 2.7	2.6 ± 2.0	4.0 ± 2.8	0.17
DN4 post-COVID, score	2.2 ± 2.2	2.4 ± 2.4	3.8 ± 1.8	0.12
ΔDN4, score	-0.3 ± 2.2	-0.2 ± 2.2	-0.2 ± 2.9	0.99

Numeric variables are summarized as means ± standard deviation. Variables were compared using one-way ANOVA. Variables that were significantly different between the no COVID-19, COVID-19, and long-COVID group were denoted as ‡*P* ≤ 0.05, †*P* ≤ 0.01, ††*P* ≤ 0.001, †††*P* ≤ 0.0001. P value for COVID-19 vs. long-COVID. Abbreviations: Δ = difference, CNFD = corneal nerve fiber density, CNBD = corneal nerve branch density, CNFL = corneal nerve fiber length, and VPT = vibration perception threshold.

### Differences in change in clinical and neuropathy measures between non-COVID-19 and COVID-19

Changes in clinical and neuropathy measures were comparable between patients who did and did not develop COVID-19. In an additional analysis, the COVID and severe COVID groups were combined to increase the sample size and also showed any statistically significant differences in clinical, or neuropathy measures compared to the no-COVID group.

### Differences in change in clinical and neuropathy measures between COVID-19 and severe COVID-19

Changes in clinical and neuropathy measures were comparable in the severe COVID-19 group compared to the no COVID-19 and COVID-19 groups. However, changes in LDL (-0.43 ± 0.96 vs. 0.33 ± 0.71, *P* ≤ 0.05) in the severe COVID-19 were significantly different compared to the COVID-19 group.  

### Differences in change in clinical and neuropathy measures between patients who did and did not develop long-COVID

Triglyceride levels increased significantly in the long-COVID group (1.05 ± 1.83) compared to the no COVID-19 group (-0.34 ± 1.26 mmol/L, *P* ≤ 0.05) and COVID-19 group (-0.25 ± 1.56 mmol/L, *P* < 0.05). Indeed, triglyceride levels were significantly higher in the long-COVID-19 group (2.82 ± 1.86) compared to the COVID-19 group (1.46 ± 0.74 mmol/L, *P* = 0.001) and no COVID-19 group (1.56 ± 0.72 mmol/L, *P* ≤ 0.01). Changes in LDL (-0.46 ± 0.76 vs. 0.33 ± 0.71, *P* ≤ 0.05) in the long-COVID group were significantly different compared to the COVID-19 group. There was a significant reduction in body weight in the long-COVID group (-8.3 ± 15.7 kg) compared to the no COVID-19 group (-4.0 ± 9.5 kg, *P* ≤ 0.01) and COVID-19 group (-0.8 ± 7.8 kg, *P* < 0.01). There were no significant differences in change in neuropathy measures in the long-COVID group compared to the no COVID-19 and COVID-19 groups.

### Change in neuropathy measures in type 2 diabetes in relation to COVID-19

There was a significant reduction in CNFD (*P* < 0.05-0.0001), CNBD (*P* < 0.01-0.0001), and CNFL (*P* < 0.01) in both the no COVID-19 and COVID-19 groups. In the patients who developed severe COVID-19, there was a significant reduction in CNBD (*P* < 0.01) and a non-significant trend for reduction in CNFL (*P* = 0.06). In the long COVID group, there was no significant reduction in any of the corneal nerve measures. There was no significant change in the prevalence of DPN defined by DN4, VPT or CNFL between the no COVID-19, COVID-19, severe COVID-19, and long-COVID groups (*P* = 0.15–0.89) (Table 5). Logistic regression showed no significant association between COVID-19 and DPN in the post-COVID phase (OR = 1.94, 95% CI: 0.65–5.81, *P* = 0.24). Similarly, linear regression models found no significant effect of COVID-19 on changes in neuropathy measures (*P* = 0.24–0.96) (Supplementary Table 1), supporting our comparative analyses.

**Table 5:** Comparison of neuropathy characteristics between pre-COVID-19 (baseline) and post-COVID-19 (follow-up visit) in participants with type 2 diabetes without COVID-19 infection, with COVID-19, severe COVID-19, and long COVID.

		Pre-COVID	Post-COVID	*P* value
No COVID-19 (*n* = 41)	DPN (DN4 & CNFL), n (%)	5 (12.2)	10 (24.4)	0.15
	DPN (DN4 & VPT), n (%)	5 (12.5)	3 (7.5)	0.46
	CNFD, fibers/mm^2^	26.3 ± 9.5	22.3 ± 8.3↓	**< 0.0001**
	CNBD, branches/mm^2^	60.2 ± 34.9	38.0 ± 27.9↓	**< 0.0001**
	CNFL, mm/mm^2^	17.3 ± 6.4	14.9 ± 6.2↓	**< 0.01**
	VPT, V	9.6 ± 5.7	9.8 ± 6.9	0.82
	DN4, score	2.5 ± 2.7	2.2 ± 2.2	0.32
COVID-19 (*n* = 21)	DPN (DN4 & CNFL), n (%)	2 (9.5)	4 (20.0)	0.34
	DPN (DN4 & VPT), n (%)	2 (9.5)	2 (9.5)	1.00
	CNFD, fibers/mm^2^	27.4 ± 9.6	23.0 ± 7.9↓	**< 0.05**
	CNBD, branches/mm^2^	70.0 ± 49.3	37.7 ± 25.4↓	**< 0.01**
	CNFL, mm/mm^2^	18.1 ± 6.8	14.6 ± 5.2↓	**< 0.01**
	VPT, V	7.8 ± 4.3	8.9 ± 5.3	0.25
	DN4, score	2.6 ± 2.0	2.4 ± 2.4	0.63
Severe COVID-19 (*n* = 8)	DPN (DN4 & CNFL), n (%)	2 (28.6)	3 (37.5)	0.71
	DPN (DN4 & VPT), n (%)	1 (14.3)	2 (25.0)	0.61
	CNFD, fibers/mm^2^	24.4 ± 8.4	21.5 ± 10.8	0.33
	CNBD, branches/mm^2^	62.0 ± 31.9	31.8 ± 15.4↓	**< 0.01**
	CNFL, mm/mm^2^	16.4 ± 6.2	13.2 ± 5.3	0.06
	VPT, V	10.5 ± 5.7	11.6 ± 6.8	0.31
	DN4, score	3.3 ± 2.5	3.1 ± 2.7	0.89
Long COVID (*n* = 9)	DPN (DN4 & CNFL), n (%)	1 (11.1)	3 (33.3)	0.26
	DPN (DN4 & VPT), n (%)	2 (22.2)	2 (22.2)	0.89
	CNFD, fibers/mm^2^	24.4 ± 5.3	25.2 ± 5.5	0.76
	CNBD, branches/mm^2^	52.1 ± 21.8	38.5 ± 18.8	0.14
	CNFL, mm/mm^2^	15.8 ± 4.5	14.9 ± 3.7	0.65
	VPT, V	13.4 ± 6.2	28.7 ± 27.2	0.30
	DN4, score	4.0 ± 2.8	3.8 ± 1.8	0.82

Categorical and numeric variables are summarized as n (%) and means ± standard deviation, respectively. Categorical variables were compared using χ^2^ and numeric variables were compared using paired t-test. Abbreviations: CNFD = corneal nerve fiber density, CNBD = corneal nerve branch density, CNFL = corneal nerve fiber length, and VPT = vibration perception threshold.

## Discussion

This study investigated the risk factors for COVID-19 and its impact on diabetic peripheral neuropathy (DPN) in patients with type 2 diabetes (T2D). A lower systolic blood pressure was associated with COVID-19, and higher weight, LDL, and vibration perception threshold (VPT) were predictors of long-COVID. Furthermore, the prevalence of long-COVID was ~ 8-fold higher in women than in men. The presence of DPN and severity of neuropathic symptoms and deficits had no association with the development and severity of COVID-19 or long-COVID. Increased triglycerides and reduced body weight and LDL were associated with long-COVID, while reduced LDL was associated with severe COVID-19.

Previous studies have shown that DPN is a risk factor for severe COVID-19^2^. However, our study found no association between the presence and severity of DPN and the development, severity, or long-term sequelae of COVID-19. A previous study showed that a significant proportion of those with COVID-19 experienced neuropathic pain or numbness within 3-months of COVID-19, particularly those with neuropathy^14^. Odriozola et al.^15^ reported that severe COVID-19 was associated with more neuropathic symptoms and widespread sensory impairment characterized by abnormal vibration and warm perception thresholds in the feet, as well as abnormal heat pain perception on the face in patients with diabetes within 3-weeks of the onset of COVID-19. In contrast, we show that COVID-19 had no impact on neuropathic symptoms (DN4) or vibration perception thresholds, which may be attributed to less severe COVID-19 as none of our patients even with severe COVID-19 required admission to the ICU. This was further supported by regression analyses, which confirmed no significant association between COVID-19 and neuropathy measures. Previously, Bitirgen et al.^16^ reported corneal nerve loss and increased corneal dendritic cells in patients with long-COVID within 4-months of COVID-19. In the current study, we found no association between the presence and severity of COVID-19 or long-COVID with corneal nerve morphology, however, CCM was undertaken almost a year after the diagnosis of COVID-19, which may have allowed time for nerve repair. Moreover, there was progressive corneal nerve loss irrespective of COVID-19, severity of COVID-19, or the development of long-COVID, suggesting a broader impact of the pandemic in relation to inadequate healthcare access due to lockdowns. Progressive corneal nerve loss might also be attributed to diabetes and its comorbidities^23^.

Our study found that elevated HDL and LDL were associated with severe COVID-19, and elevated LDL was associated with long-COVID. A previous study showed that both low and high LDL levels were associated with severe COVID-19^24^. In contrast, another study reported that low LDL, HDL, and cholesterol at hospital admission were associated with severe COVID-19 and increased mortality whilst high HDL was associated with a lower risk of COVID-19 infection^25^, suggesting a potential protective role of HDL. Hence, the association of elevated HDL and LDL with severe COVID-19 and long-COVID warrants further research. This study also showed that low systolic blood pressure was associated with an increased risk of COVID-19. A previous study showed that hypertension was associated with severe COVID-19, but a systolic blood pressure less than 90 mmHg was associated with increased COVID-19 mortality^26^. A previous study has also shown that ICU admission or death was associated with a BMI above 23 kg/m^2^.^27^ This study showed no association between the severity of COVID-19 and body weight but did show that higher body weight was associated with long-COVID and those with long-COVID lost an excessive amount of weight, perhaps reflecting the morbidity associated with this condition. A previous study found no association between body weight and long-COVID risk^28^, underscoring the need for more research into how body weight and BMI might affect the outcomes of COVID-19.

Our study found an 8-fold increase in the prevalence of women who developed long-COVID, consistent with a previous study showing a 4-fold increase^29^. In the US, around 8% of the population is affected by autoimmune diseases, with women making up 78% of these cases^30^ and as viruses can trigger autoimmune disease, it is relevant that long-COVID has been suggested to be an estrogen-associated autoimmune condition^31^.

We acknowledge a major limitation is the small sample size which may have compromised the statistical power and restricts the generalizability of the results. Furthermore, the follow-up assessments were undertaken long after the initial onset of COVID-19 and may therefore have missed the initial impact of COVID-19. We also recognize that some participants deemed not to have COVID-19 may have been asymptomatic and therefore had not been tested for COVID-19.

In conclusion, this study suggests that low systolic blood pressure, altered lipids, body weight, higher VPT, and gender may determine the impact of COVID-19 in patients with T2D. There was no evidence of an adverse effect of COVID-19 on neuropathy, which may be attributed to recovery almost 11 months after the initial infection.

## Electronic supplementary material

Below is the link to the electronic supplementary material.

Supplementary Material 1

## Data Availability

The data that support the findings of this study are available on request from the corresponding author.
