# Supplementary material for: COVID-19 and neuropathy in type 2 diabetes
Source: Sci Rep. 2025 Apr 1;15:11188. doi: 10.1038/s41598-025-95133-4 (PMC11961607; doi:10.1038/s41598-025-95133-4)
Supplement: Supplementary file 1 — Supplementary Material 1 [file 41598_2025_95133_MOESM1_ESM.docx]

Supplementary Table 1. Logistic and linear regression analyses assessing the association between COVID-19 and neuropathy measures in patients with type 2 diabetes.

| Dependent variable | Odds Ratio (OR) | 95% CI for OR | P value |
| --- | --- | --- | --- |
| Diabetic peripheral neuropathy | 1.94 | 0.65 to 5.81 | 0.24 |
| Dependent variable | Unstandardized β coefficient | 95% CI | P value |
| ΔCNFD, fibers/mm^2^ | 1.04 | -2.39 to 4.47 | 0.55 |
| ΔCNBD, branches/mm^2^ | -6.14 | -21.90 to 9.62 | 0.44 |
| ΔCNFL, mm/mm^2^ | -0.58 | -2.84 to 1.69 | 0.61 |
| ΔVPT, V | 0.08 | -2.58 to 2.73 | 0.96 |
| ΔDN4, score | -0.03 | -1.07 to 1.01 | 0.96 |

Abbreviations: Corneal nerve fiber density (CNFD), corneal nerve branch density (CNBD), corneal nerve fiber length (CNFL), and vibration perception threshold (VPT).
